# A smallest 6 kda metalloprotease, mini-matrilysin, in living world: a revolutionary conserved zinc-dependent proteolytic domain- helix-loop-helix catalytic zinc binding domain (ZBD)

**DOI:** 10.1186/1423-0127-19-54

**Published:** 2012-05-29

**Authors:** Wei-Hsuan Yu, Po-Tsang Huang, Kuo-Long Lou, Shuan-Su C Yu, Chen Lin

**Affiliations:** 1 Institute of Biochemistry and Molecular Biology, College of Medicine, National Taiwan University, Ren-Ai Road, Taipei, Taiwan; 2 Graduate Institute of Oral Biology, College of Medicine, National Taiwan University, Ren-Ai Road, Taipei, Taiwan; 3 NTU-DRCP Lectures and Core for Membrane Proteins, Center for Biotechnology, National Taiwan University, Chang Sing Street, Taipei, Taiwan; 4 Institute of Biotechnology, National Taiwan University, Chang Sing Street, Taipei, Taiwan

**Keywords:** Matrilysin, Zinc-dependent proteolytic domain, Catalytic zinc binding domain, Helix-loop-helix, SC44463

## Abstract

****Background**:**

The Aim of this study is to study the minimum zinc dependent metalloprotease catalytic folding motif, helix B Met loop-helix C, with proteolytic catalytic activities in metzincin super family. The metzincin super family share a catalytic domain consisting of a twisted five-stranded β sheet and three long α helices (A, B and C). The catalytic zinc is at the bottom of the cleft and is ligated by three His residues in the consensus sequence motif, HEXXHXXGXXH, which is located in helix B and part of the adjacent Met turn region. An interesting question is - what is the minimum portion of the enzyme that still possesses catalytic and inhibitor recognition?”

****Methods**:**

We have expressed a 60-residue truncated form of matrilysin which retains only the helix B-Met turn-helix C region and deletes helix A and the five-stranded β sheet which form the upper portion of the active cleft. This is only 1/4 of the full catalytic domain. The E. coli derived 6 kDa MMP-7 ZBD fragments were purified and refolded. The proteolytic activities were analyzed by Mca-Pro-Leu-Gly-Leu-Dpa-Ala-Arg-NH2 peptide assay and CM-transferrin zymography analysis. SC44463, BB94 and Phosphoramidon were computationally docked into the 3day structure of the human MMP7 ZBD and TAD and thermolysin using the docking program GOLD.

****Results**:**

This minimal 6 kDa matrilysin has been refolded and shown to have proteolytic activity in the Mca-Pro-Leu-Gly-Leu-Dpa-Ala-Arg-NH2 peptide assay. Triton X-100 and heparin are important factors in the refolding environment for this mini-enzyme matrilysin. This minienzyme has the proteolytic activity towards peptide substrate, but the hexamer and octamer of the mini MMP-7 complex demonstrates the CM-transferrin proteolytic activities in zymographic analysis. Peptide digestion is inhibited by SC44463, specific MMP7 inhibitors, but not phosphorimadon. Interestingly, the mini MMP-7 can be processed by autolysis and producing ~ 6 ~ 7 kDa fragments. Thus, many of the functions of the enzyme are retained indicating that the helix B-Met loop-helix C is the minimal functional “domain” found to date for the matrixin family.

****Conclusions**:**

The helix B-Met loop-helix C folding conserved in metalloprotease metzincin super family is able to facilitate proteolytic catalysis for specific substrate and inhibitor recognition. The autolysis processing and producing 6 kDa mini MMP-7 is the smallest metalloprotease in living world.

## **Background**

**Figure 1:**
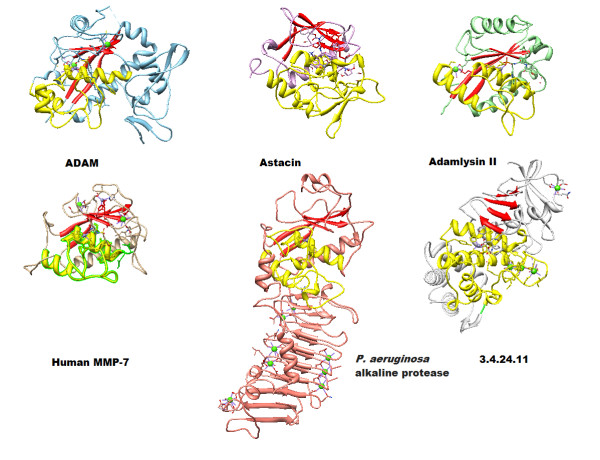
**Structure comparison of Human MMP7 with metalloproteases.** Details of the PDB structure codes of related proteins are as followed: ADAM: 2RJP, Astacin: 1QJI, Adamlysin II: 2AIG, Human MMP-7: 1MMR, P. aeruginosa alkaline protease: 1AKL, 3.4.24.11 protease: 1THL. It is interesting to notice the locations of the catalytic domain consisting of a red twisted β sheet as TAD and long yellow α helices which zinc-binding motif located on. All the structures are viewed in approximately the same orientation for better comparisons. TADs are presented in red ribbons, and catalytic HLH were presented in yellow ribbon. The main chains of the six metalloproteases are represented with ribbons in different colors. The Important motifs, such as calcium, zinc and their binding residues, and inhibitors are presented with ball-and-stick style. The side-chains of crucial basic residues discussed in the text are shown as colored sticks.

Matrix metalloproteases, or matrixins, are a family of calcium and zinc-dependent metalloenzymes that degrade a great variety of extracellular matrix components. The matrixins are part of a large family, the “metzincin” family, which includes Family M10 (matrixins and serralysins) and Family M12 (astacins and reprolysins). All four groups share the consensus zinc-binding sequence and Met-turn, which forms the base of the binding pocket. These 4 groups are not closely related by sequence identity, and it is their protein fold that first led to their assignment to a single metzincin group. The active centers have in common the characteristic HEXXHXXGXXH zinc-binding motif located in helix-2 (Table 1) [1]. Three histidine residues serve as zinc ligands, and the glutamic acid residue polarizes a water molecule involved in the nucleophilic attack on the scissile peptide bond. The mutation of this glutamic acid residue in MMP-7 can lead to reduction of specific activity up to 1000-fold [2]. Another interesting feature is the integrity of the 12 Å wide catalytic groove produced by the combination of the five –stranded α sheet, the catalytic β helix II, and the Met-turn loop (Figure 1). Instead of using cysteine disulfide bridging, two calcium and one or two structural zinc atoms are required to maintain the correct architecture. Two homologous EF-hand calcium-binding motifs and the triple His zinc-binding motif stabilize the five-stranded β sheet [3]. The third calcium in the X-ray crystal structure of the TIMP-1/MMP-3 complex was located adjacent to the Met-turn [4,5]. The specific importance of this structural calcium is still unrevealed. The fourth-strand of this β sheet contributes the major contact area for docking the natural MMP inhibitors, the TIMPs [6,7].

**Table 1:** Sequence alignment of two representatives of each subfamily of the metzincins

	Zinc-binding Signature	Met-turn
ADAM	TAAHQLGHVFNMLHDNSK	PLSTSRHVMAPVMAHVD
Astacin	TIIHELMHAIGFYHEHT	EDYQYYSIMHYGKYSFS
Adamalysin II	TMAHELGHNLGMEHDGK	LRGASLCIMRPGLTPGR
Matrilysin	AATHELGHSLGMGHSSD	HSSDPNAVMYPTYGNGD

Only the sections containing the zinc-binding histidine residues and the Met-loop areshown. The histidine zinc-ligands, the catalytically active glutamic acid residue, the residue directly following the third His ligand, the conserved Met residue, and the two positions after the Met are in.

a *Pseudomonas aeruginosa* alkaline proteinase.

b 3.4.24.11 protease.

PDB code: (ADAM: 2RJP[20], Astacin: 1QJI[21], Adamlysin II: 2AIG[22], HumanMMP-7: 1MMR[3], P. aeruginosa alkaline protease: 1AKL[23], 3.4.24.11 protease: 1THL[10].)

An interesting question is - what is the minimum portion of the enzyme that still possesses catalytic activity?” It is known that in most MMPs, not only the propeptide but also the hemopexin domain can be deleted without effect on activity (e.g., stromelysin). Matrilysin, MMP-7, is already “truncated”, and the active form consists only of a 180 residue catalytic domain. However, in the gelatinases this catalytic domain is divided by the insertion of 3 fibronectin-like repeats. The division leaves the active zinc and sixty residues on the carboxyl side of the ‘break’. We decided to employ recombinant protein techniques to generate the truncated form of rat matrilysin which is lacking the five-stranded β sheet (N-domain) and to address the following questions concerning the resulting 60-mer. First, is the helix B-Met-turn-helix C the minimum domain required for activity; second, does this minimal domain still possess the original substrate recognition; and third, in the absence of the five-stranded-β-sheet, can matrilysin complex with its native inhibitor, TIMP, and synthetic inhibitors?

## **Methods**

### **Expression and purification of recombinant rat matrilysin zinc-binding domain (ZBD) domain proteins in*Escherichia coli*BL 21(DE3) cells**

The rat cDNA containing zinc-binding domain (a.a. 212–267) insert corresponding to human cDNA containing zinc-binding domain (a.a. 188–247) of rat matrilysin was synthesized by using a pair of primers, Zn-UMP 5’TCACAT**ATG**GGAGTGAACTTCCTGTTT3’ and the Sp6 primer which is from the SP6 promoter region of pGEM3Zf(+) in one-step PCR and sub cloned into the Nde1 and BamH1 sites of PET3a vector (Novae). The cDNA insert of the zinc-binding domain (ZBD) expression construct was completely sequenced by using the Sequenase Version 2.0 Kit (U.S. Biochemical Corp.). The expression plasmid was used to transform *E. coli* BL21 (DE3) cells (Novagen). Cells were cultured for 6 h (OD600 = 0.2 ~ 0.4) followed by 2 hrs with IPTG. Cells were collected, passed through a French press and centrifuged at 13,000 rpm for 20 mines. The pellets were washed 3X with 10 ml of inclusion-body wash solution (0.01% Triton X-100, 50 mM Tris, pH7.5). The pellets were suspended in 8 M urea, 0.2 M NaCl, 50 mM Tris, pH 7.5, 0.02% Na azide. After 48 hrs at 4 °C, the sample was centrifuged. The soluble fraction was fractionated by passing through a P30 molecular sieve chromatography column (Pharmacia) in 8 M urea, 0.2 M NaCl, 50 mM Tris, pH 7.5, 0.02% Na azide to partially separate the high molecular weight and the 6 kDa ZBD, and then the fractions contained 6 kDa ZBD were combined and applied to a 2 ml zinc chelate-Sepharose 6LB column (Pharmacia). This was washed with 30 ml buffer containing 8 M urea, 0.2 M Nacl, 50 mM Tris, pH 7.5, 0.02% Na azide. The collected fractions were subjected to high resolution SDS-PAGE analysis and followed by silver staining.

### **High resolution SDS-PAGE**

The stock solutions for high resolution SDS-PAGE were prepared as follows: Acryl/Bis (100 ml) contains 25.0 g acrylamide and 0.25 g Bis; lower buffer (100 ml) contains 12.1 g Tris (1 M), 3.76 g Glycine (0.5 M), 2.0 g SDS (4%), pH 9.0; upper buffer (25 ml) contains 1.51 g Tris, pH 6.7, 1.4 ml 0.5 M EDTA, and 0.7 g SDS; running buffer (300 ml) contains 3.63 g Tris, 3.38 g Glycine, 0.3 g SDS, pH 8,45, self-set, pre-SDS. For resolving the molecular weight ranging between 2–100 kDa proteins, a 15% high resolution running gel was prepared by mixing 6.0 ml Acryl/lBis, 2.0 ml lower buffer, 1.0 ml H_2_O, 1.0 ml 50% Glycerol, 10 μl; TEMED, 100 μl; 10% APS. The mixture with running gel solution was poured into the gel cast, leaving a 2.5 cm space from the top for 30 minutes. The stacking gel solution containing a mixture of 1.6 ml Acryl/Bis, 1.4 ml upper buffer, 6.0 ml H2O, 1.0 ml 50% Glycerol, 15 μl TEMED, 100 μl 10% APS was poured. The stacking gel was polymerized for another 30 minutes. Samples were loaded into the wells and run at 90 V in the presence of full strength running buffer in the upper chamber, and 1; 1 dilution of running buffer in the lower chamber.

### **Refolding of zinc binding domain**

The purified and denatured (8 M urea) 6 kDa ZBD was diluted drop wise 10-fold in ice-cold refolding buffer (20 mM Acetate, pH 5.6, 10 mM CaCl2, 1 μm ZnCl2, 0.2 mg/ml heparin and 0.05% Triton X-100). After 30 minutes, the sample was centrifuged at 14,000 rpm for 10 minutes to remove the insoluble portion. Storage of soluble refolded protein was at −70 °C in 18% Glycerol.

### **Western blot**

The protein samples were separated by SDS-PAGE, transferred to nitrocellulose membranes (BioRad). Then 0.1% Tween-20 (TTBS) containing 5% non-fat milk was used to block the membrane for 3 hrs at 24 °C. The first antibodies (e.g., RM7-C and RM7-P) were applied at 4 °C overnight. After 3X washing with TTBS/milk, the second antibody (e.g., goat anti-rabbit IgG-alkaline phosphatase) was applied for 2 hrs. Then the transblot was washed 3X with TTBS/milk and stained with NBT/BCIP (Pierce).

### **Mca-peptide assay**

For assay the proteolytic activities for the E Coli derived purified refolded recombinant mini-Matrilysin(ZBD) proteins, Enzyme assays were conducted at 37 °C using 50 mM Tris–HCl buffer, pH 7.5, containing 100 mM NaCl and 10 mM CaCl2,1nM Zinc Chloride, 0.1% tween 20 plus Brij 35 in a total volume of 2.5 ml. The substrate, Mca-Pro-Leu-Gly-Leu-Dpa-Ala-Arg-NH2 (λex = 380 nm, λem = 460 nm) was first dissolved in DMSO and the solution was diluted to give a final concentration of 0.1 mM, using the buffer described above. The fluorescence of 7-amino-4-methyl-coumarin produced was monitored using a Hitachi fluorescence spectrofluorimeter, model MPF-2A, equipped with a recorder. The measurements were carried out with excitation at 380 nm and emission at 460 nm. The fluorescence spectrofluorimeter was set to zero with substrate in assay buffer. For each enzyme, the initial rate of cleavage of Mca-Pro-Leu-Gly-Leu-Dpa-Ala-Arg-NH2, measured over 10 to 15 minutes, Stopped assays were made with the 0.1 M sodium acetate, pH 4.0 at different time points of interest [8]. The fluorescence spectrofluorimeter was standardized so that at 10 μM solution of MCA in 0.1% DMSO gave 1.0 relative fluorescence unit. 7-amino-4-methyl-coumarine is used as the reference compound to make a standard curve to quantitate the hydrolysis products from the reactions. The H-Pro-Phe-Arg-AMC peptide is a non-cleavable substrate as negative background control to standardize the instrument.

### **Amc-peptide assay**

Enzyme assays were conducted at 37 °C in 50 mM Tris–HCl buffer, pH 7.5, containing 100 mM NaCl and 10 mM CaCl2 in a total volume of 2.5 ml. The substrate, H-Pro-Phe-Arg-AMC was first dissolved in DMSO and the solution was diluted to give a final concentration of 0.1 mM in the buffer described above. The reaction was started by the addition of 10 μl of enzyme and the fluorescence of 7-amino-4-methyl-coumarin produced was monitored using a fluorescence spectrofluorimeter. The measurements were carried out with excitation at 380 nm and emission at 460 nm. The fluorescence spectrofluorimeter was standardized so that at 10 μM solution of MCA in 0.1% DMSO gave 1.0 relative fluorescence unit. 7-amino-4-methyl-coumarine is used as the reference compound to make a standard curve to quantitate the hydrolysis products from the reaction.

### **N-TIMP-1 and synthetic inhibitors inhibition assay**

Synthetic fluorogenic peptide substrate, Mca-Pro-Leu-Gly-Leu-Dpa-Ala-Arg-NH2, for MMP-7 [8] was used to assay the ability of N-TIMP-1 and several synthetic inhibitors, BB94 and SC44463 to inhibit recombinant ZBD. Assay of the inhibition of recombinant ZBD and human active MMP-7 by recombinant full length TIMP-1, N-TIMP-1(gift from Dr. Keith Brew [6,9]) and synthetic inhibitors was carried out by pre incubating the MMP (0.1 to 1 nM) and TIMP (a series of concentrations) in 50 mM Tris–HCl, pH 7.5, containing 0.15 M NaCl, 10 mM CaCl2 and 0.02% Brij at 37 °C for 1 hr. The time required for equilibration was determined by following the progress of inhibition after mixing MMPs and TIMP at concentrations in the range used in the assay. An aliquot (60 μl) of substrate (15 μM) was then added to 540 μl of the pre incubated MMP-TIMP mixture and activity was measured at 37 °C by following product release with time.

### **CM-transferrin zymography**

CM-transferrin (bovine), transferrin was reduced and carboxymethylated as described by Nagase (1995). CM-transferrin (0.3 mg/ml) was embedded in 12.5% SDS–PAGE gel. Purified & refolded 6 kDa MMP-7 ZBD was treated with sample buffer without dithiothreitol at room temperature and electrophoreses until the dye-front was near the bottom of the gel. Each gel was washed 5 times with 50 ml 2.5% Triton X-100, 50 mM Tris, pH 7.5, 4 °C, 20 min each, to remove SDS and then 3 times with 50 ml buffer plus 5 mMCaCl2. The gel was washed with 50 ml incubation buffer (50 mM Tris, pH 7.5, 5 mM CaCl2) and then incubated in 50 ml this buffer with added protease inhibitors (50 mM each of Z-Phe-chloromethylketone, Tosyl-Phe-chloromethylketone (ZPCK) and amino ethyl benzenesulfonyl fluoride (PMSF)) for 18 h, 37 °C, with gentle shaking. Gels were stained with 0.1% Coomassie blue in 40% MeOH, 10% acetic acid, for 50 min, and destained with 7% acetic acid. The gels were scanned and digitized with a UVP analyzer and two dimensional intensities were determined with the Gelbase program (Ultra Violet Products, Upland, CA).

### **Molecular docking of SC44463, BB94 and phosphoramidon**

SC44463, BB94 and phosphoramidon docking and data analysis. BB94 is selected for positive binding and phosphoramidon for negative control on MMP7. The 3D structures of the human MMP7 protease [3] (PDB code: 1MMR) and thermolysin [10] (EC#: 3.4.24.11) ((PDB code: 1THL) were retrieved from the Protein Data Bank. PRODRG program (http://davapc1.bioch.dundee.ac.uk/ programs/prodrg/) were used to generate the coordinate and topology files of SC44463 [11,12], BB94 [13-15] and Phosphoramidon [16,17]. SC44463, BB94 and Phosphoramidon were computationally docked into the 3D structure of the human MMP7 protease and thermolysin using the docking program GOLD [18] (version 2.1.2; Genetic Optimization for Ligand Docking, CCDC, Cambridge, UK) or PPDOCK program (http://140.112.135.49/ppdock/). GOLD operates with a genetic search algorithm and allows for complete ligand and partial-binding site flexibility [18]. Because SC44463, BB94 and Phosphoramidon may be the inhibitors of the catalytic centers which zinc-binding motif located in helix-2, we defined the binding site to the inhibitor as the putative znic catalytic pocket. The results were ranked by GOLD’s scoring function which is a molecular mechanics-like function with four terms based on protein–ligand hydrogen bond energy, protein–ligand van der Waals energy, ligand internal van der Waals energy, and ligand torsional strain. The 100 steps of energy minimization of the best docking structure were carried out using the CNS programs [19]. The interactions between SC44666 and the human MMP7 protease were analyzed and illustrated by Discovery Studio 3.0 (Accelrys Software Inc.) and Chimera (The University of California).

## **Results**

### **Structure comparison of human MMP7 with metalloproteases**

We take some metalloproteases to compare with human MMP7 [3] as fallowing: ADAM [20], Astacin [21], Adamlysin II [22], *P. aeruginosa* alkaline protease [23], 2.4.24.11 protease [10]. It is clear to show that the locations of the catalytic domain consisting of a twisted red β sheet as TAD and long yellow α helices which zinc-binding motif located on (Figure 1). They have similar folding structures which are made up of two domains: an “upper” N-domain comprising the N-terminal part of the polypeptide chains forming the five-stranded β sheet and a lower C-domain form a α-helix-loop-α-helix packing. α-Helices shown in yellow, β-strands shown in red, and other parts of the polypeptide chains in colors. All six molecules are shown with the central active center cleft lying horizontally in the paper plane after superimposed for structural comparison. Six metalloproteases structure share the conserved HLH folding (domain) with different length of loops.

This stretch from residue 188–247 of human MMP7 could be considered the minimum sequence required for enzymatic processes, substrate analogue inhibitor docking, and substrate recognition. The three-dimensional structures of the adamalysin II from rattle snake venom (reprolysin), alkaline proteases from *Pseudomonas aeruginosa* (serralysin), and astacin from crayfish are topologically similar with respect to the five-stranded-β-sheet (N-domain) and three α-helices (C-domain) arranged in typical sequential order (Figure 1) [24,25].

The strands sIII and sIV (β sheets) forming the upper wall of the active cleft are quite conserved in length and position in the metzincin family [2]; the loop connecting them is quite different in all four subclass members. In the matrixins, the sIII-sIV linker exhibits an S-shape, looping around a structural zinc ion and a tightly bound calcium ion. All six enzymes show an almost identical active site environment (Figure 1) [24]. Helix hB contains the short consensus motif **HEXXH** (Table 1). The two histidine zinc ligands are separated by a single helix turn, which allows a concerted approach by two flanking imidazoles toward the catalytic zinc. The carboxylate group of the intermediate glutamic acid is involved in the fixation of a zinc-bound water molecule (Table 1, Figure 1). The active site helix of the metazincin is terminated at an invariant glycine residue, three residues away from the second histidine zinc ligand. Subsequently, another three residues after the glycine, the third histidine metal ligand is projecting toward the catalytic zinc from below. The most remarkable 1, 4 tight turn of virtually identical conformation and position relative to the catalytic zinc, called the Met-turn, appears to be essential for the structural integrity of the zinc-binding active site of the metzincin family. Following the Met-turn is the C-terminal helix hC. The helix hC is a potential amphipathic helix participating in lining the active cleft and connecting the N-domain through salt bridge formation between the Asp in the hC and Trp in the N-terminal [24,26]. Although there is an almost identical active site environment in the four groups of the metzincin family, each still exhibits distinct substrate specificity. TAD may be responsible for additional regulation and unfolding substrate for MMP7.

### **Construction, expression and purification of*E.Coli*BL21(DE3) derived recombinant 6 kDa catalytic zinc-binding domain (ZBD) proteins**

**Figure 2:**
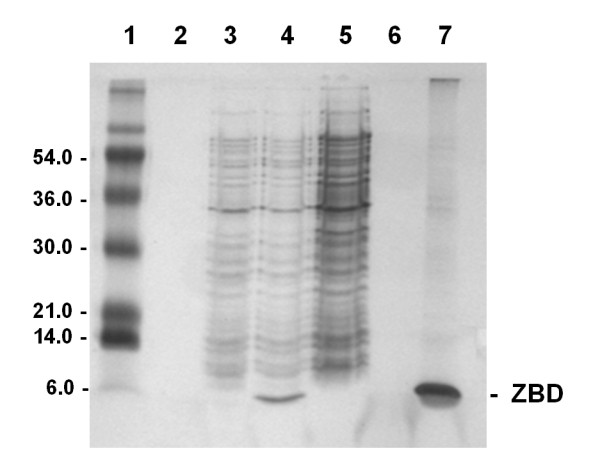
**The*E. coli*BL21 (DE3). PET3a ZBD Expression Profile of Recombinant ZBD.*** E. coli* BL21 (DE3). *PET3a* ZBD transformed cells were grown at 37 0 C to OD600 reading 0.2 ~ 0.4, 10ul of total cell extracts from 200 μl of cells solibilized in 1% SDS sample buffer as control for before IPTG induction were prepared as method described (lane 3). After 2 hour 0.8 mM IPTG induction, 10ul of total cell extracts from 100 ul of cells were prepared as after IPTG incubation (lane 4). 10ul of total cell extracts from 100 μl of 2 hours IPTG inducing PET 3a mock transformation BL21 (DE3) cells was prepared as negative control (lane 5). The pellet after French press and inclusion bodies 0.1% triton X-100 wash steps were prepared (lane 7). Lane 1: Molecular weight standard; Lane2 and 6: Blank.

**Figure 3:**
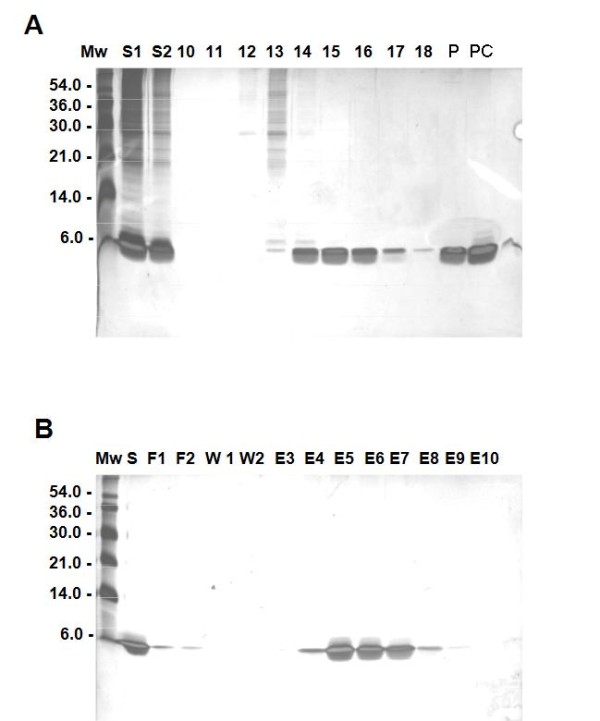
**Purification of Recombinant ZBD.*Panel A*:** shows the silver staining for the high resolution SDS-PAGE analysis for the purification fraction profile from the P30 molecular sieve chromatography. The 8 M Urea 50 mM Tris pH 7.5 solubilized inclusion bodies (lane S2) was concentrated 10 fold (laneS1) as the start material before loading onto P30 column. 15 μl of the fractions gave OD280 reading were subjected to high resolution SDS-PAGE analysis (lane 10–17). The combined fractions #14-#15 (lane p) were concentrated 10 fold (lane pc). ***Panel B*:** shows the silver staining for the high resolution SDS-PAGE analysis for the purification fraction profile from the fractions zinc-chelate chromatography. Combining the partial purified fractions containing ZBD from P30 collumn (lane S) were subjected to the zinc-chelate affinity. The unbound fall-through fractions (lane F1 and F2) and the wash buffer fractions are shown (lane W1 and W2). The bound ZBD was eluted with pH 4.5 buffer and the low pH eluted fractions (lane E3-E10).

The expected molecular weight protein of 6 kDa appears in the total extract of BL21 (DE3). PET3a.ZBD cells after 2 hour IPTG induction (Figure 2, lane 4), but not in the total cell extracts of negative control BL21 (DE3).PET3a cells after 2 hour IPTG induction (Figure 2, lane 5) or before IPTG induction of transformed cells (Figure 2, lane 3). The *E.coli* derived recombinant ZBD predominantly appeared in the insoluble fraction called inclusion bodies (Figure 2, lane7). The 8 M Urea solubilized inclusion bodies (Figure 3A, lane S2) were concentrated 10 fold (Figure 3A, lane S1) and loaded onto the molecular sieve chromatography P30 and partial separating the high molecular weight (Figure 3A, lane 12& 13) and the 6 kDa ZBD (Figure 3A, lane 14–17). In order to further purify 6 kDa ZBD, the P30 column were used and the fractions containing 6 kDa ZBD were pooled together (Figure 3B, lane S) and then applied to a 2 ml zinc chelate-Sepharose 6LB column (Pharmacia). In the fall-through from the zinc column fractions, there is a trace of a 6 kDa bacterial protein (Figure 3B, lane F1 and F2). Washing with 30 ml column-wash buffer was followed by elution of the bound ZBD with pH 4.5 buffer (Figure 3B, lane E3-E8). Greater than 95% purity of recombinant ZBD was shown in the silver stained high resolution SDS-PAGE.

### **Western blot assay for recombinant 6 kDa ZBD**

**Figure 4:**
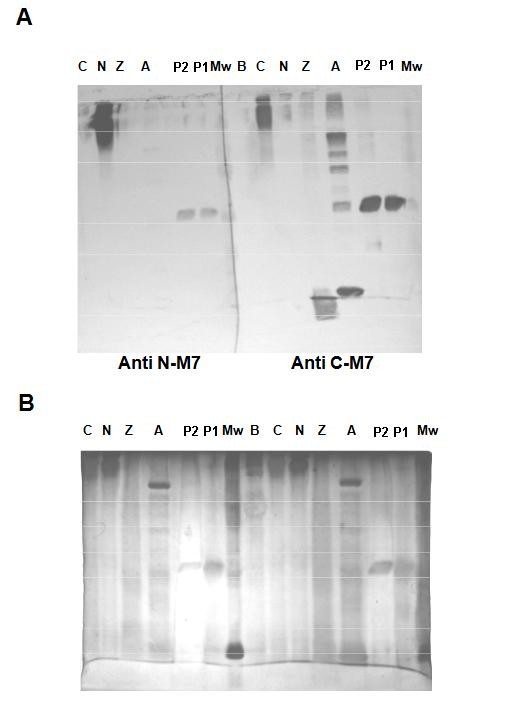
**Western Blot Analysis for ZBD.** The polyclonal antibodies, anti-propeptide (RM7-P) and anti-C-terminal peptide (RM7-C), were used to detect the final purified fraction of ZBD. ***Panel A left***: The transblot was reacted with RM7-P anti serum which specifically recognizes the proenzyme (lane P1 and P2) and the peptide22-34 conjugated BSA (lane N); ***Panel A right*** : The transblot was reacted with RM7-C anti serum which recognizes all populations of recombinant MMP-7 and the peptide238-247 conjugated BSA (lane C), except for the peptide22-34 conjugated to BSA (lane N). Both antibodies have no cross-reactivities for the BSA carrier (laneB) or each other. ***Panel B***: The silver staining for the high resolution SDS-PAGE shows the sample loading profile. Mw: Molecular weight standard; P1 and P2: proMMP-7; A: all populations of activated MMP-7. Z: recombinant CZBD; N: peptide22-34 conjugated BSA; C: the peptide238-247 conjugated BSA. B: BSA.

The molecular weight 6 kDa deduced from 60 amino acids in this fragment of the purified ZBD was further confirmed by western blot with anti-MMP-7 peptide255-267(RM7-C) and anti-MMP-7 peptide43 55(RM7-P) polyclonal antibodies [27]. Each polyclonal antibody showed no cross-reaction with the opposite peptide conjugated to BSA (Figure 4A, lane N & C) or the BSA carrier (Figure 4B, lane B). This purified 6 kDa zinc binding region of rat matrilysin can only react with anti-MMP-7 peptide255-257 polyclonal antibody (RM7-C) (Figure 4A, right panel lane Z), but not anti-MMP-7 peptidere43-55(RM7-P) (Figure 4A, left panel lane Z). This immunodetection confirmed that the zinc-chelate affinity-purified 6 kDa protein is the expected recombinant ZBD protein and the fact that there is no trace of cross-reactivity shown by using anti-MMP-7 peptide43-55 indicates that 6 kDa proteins are not derived from the N-terminal part of rat matrilysin, instead of being produced by autolysis post activation (Figure 4A, right panel lane A).

### **Triton X-100 and heparin are accessory factors folding recombinant ZBD in vitro**

**Figure 5:**
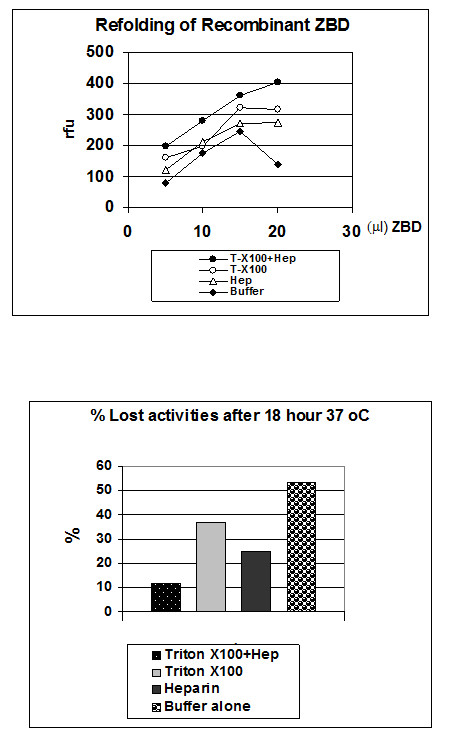
**Combination of 0.05% Triton and 0.2 mg/ml heparin give the optimal refolding activities to cleave the synthetic coumarin-labelled peptide substrate, Mca-Pro-Leu-Gly-Leu-Dpa-Ala-Arg-NH2.*Panel A*:** Shows the refolded ZBD activities increased in dose-dependent manner. In the absence of the refolding accessory factors, Triton X-100 and heparin. The significant reduced activities in the high-concentration (> 100 μg/ml) was observed which could be due to autolysis. ***Panel B*:** Under 37 °C incubation for 18 hours, Triton X-100 and heparin can prevent the activity loss. (All experiments were repeated at two batch of purification and refolding preparation and data collected from a representative experiments).

Unlike the inclusion bodies of recombinant proMMP-7 or active MMP-7 which can be partially purified by several washing with Tris 50 mM pH 7.5, 0.25% Triton X-100 and 5 mM DTT, the inclusion bodies of recombinant ZBD are easily solubilized by 0..25% Triton X-100 and demonstrate MMP activity (data not shown). This is quite a unique property of the inclusion bodies of recombinant ZBD. In most cases of recombinant proteins over-expressed in BL21 (DE3), it usually requires strong chaotropic reagent, such as 4 ~ 8 M Urea or 4 M Guanidine, to solubilize the inclusion bodies. Surprisingly, we found that 0.05% Triton X-100 is enough to solubilize the inclusion bodies of recombinant ZBD (data not shown). This raised the possibility that Triton X-100 might also be an accessory factor in the folding process of ZBD, especially since it is frequently added to various MMPs as a stabilizer. Interestingly, 0.25% Triton X-100 had been used in the zymography procedure to remove the SDS and assist folding in gels. My previous studies also showed that heparin can enhance the activities of MMPs by stabilizing the enzymes. The result from the refolding test showed that Triton X-100 was a better accessory folding factor than heparin, but the combination of 0.05% Triton X-100 and 0.3 mg/ml heparin gave the best refolding activities in the Mca-peptide assay (Figure 5A). The instability appeared to be the major factor concerned in the refolding process, because in the absence of the accessory folding factor when the concentration of ZBD reached 40 ng/per assay, the activities of ZBD decreased dramatically. In the next test, after challenging with 18 hrs 37 °C pre incubation, the loss of activity of refolded enzyme for each refolding condition was measured by the Mca-peptide assay. There is only 13% of total activity lost in the presence of Triton X-100 and heparin. Heparin seemed to be a more important factor and did a better job than Triton X-100, 23% vs 37% lost of total activity (Figure 5B). In the absence of both factors, 52% of total activity was lost after 18 hrs 37 °C pre incubation (Figure 5B).

### **The helix-loop-helix, 6 kDa ZBD, provides sufficient structure for substrate and inhibitor recognitions**

**Figure 6:**
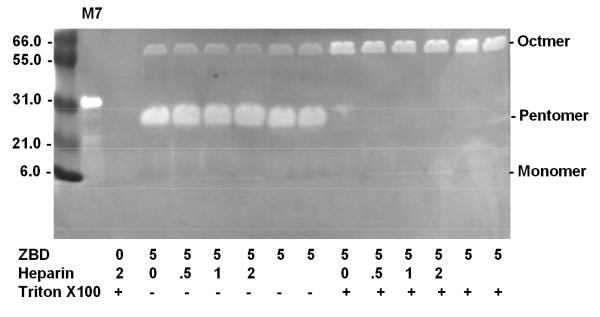
**Mca-Pro-Leu-Gly-Leu-Dpa-Ala-Arg-NH2 assay for characterization of refolded ZBD.*Panel A*:** Under the optimized conditions, the refolded ZBD shows increasing enzymatic activity in dose-dependent manner. No significant activity loss was found in the high concentration situation. ***Panel B*:** approximately 6 ng/ml refolded ZBD shows the increasing activity during the time course study and no significant activity loss during overnight incubation. ***Panel C*:** Recombinant ZBD can be inhibited by 10 nM EDTA, 1 mM CoCl2 and synthetic inhibitors, 50 nM BB94 & SC44463 and CoCl2, but not b6 250 nM Phosphoramidon. (All experiments were repeated at two batch of purification and refolding preparation and data collected from a representative experiments).

**Figure 7:**
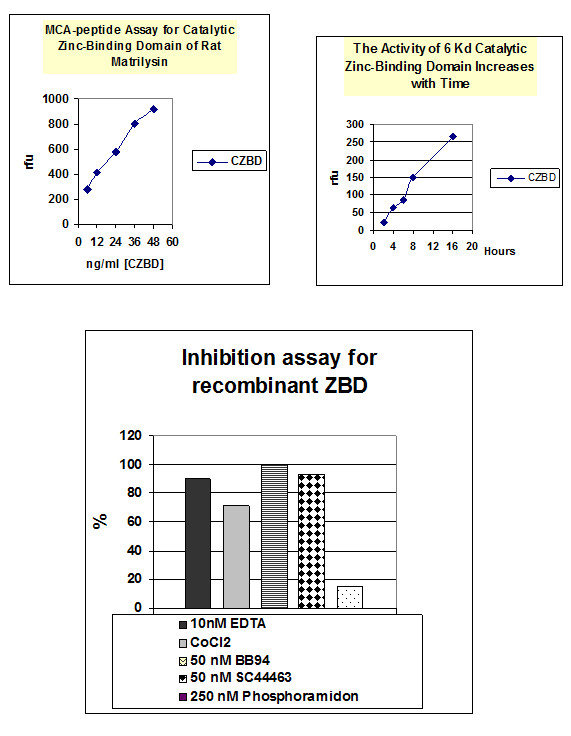
**The polymerization of the 6 kDa ZBD of MMP-7 in pentamer and Octamer demonstrate the significant proteolytic activities towards to the CM-transferrin substrate in CM-transferrin zymographic assay.** 300 μg of craboxylmethylated transferrin (CM-transferrin) was co-polymerized with SDS-PAGE as a substrate gel for analyzing the MMP-7 activities in situ.

The activity was determined by hydrolysis of the coumarin labeled-peptide, Mca-Pro-Leu-Gly-Leu-Dpa-Ala-Arg-NH2 which has been reported to be a very sensitive substrate for matrilysin. Another peptide substrate, H-Pro-Phe-Arg-AMC, which has been reported a sensitive substrate for plasminogen was used to test the substrate specificity of 6 kDa ZBD. The results revealed that the activities are increasing in dose-dependent manner against Mca peptide (Figure 6A), but not Amc-peptide (data not shown). The buffer alone or enzyme itself did not give any significant intrinsic fluorescence reading. The *k*_*cat*_/*K*_*m*_ is about 10^6^ M^-1^ s^-1^determined by hydrolysis of Mca-peptide substrate, which is about 400 ~ 500 fold less than the wild type activity. Interestingly, according to the X-ray crystal structure even though there is no the third calcium revealed in the catalytic zinc-binding domain, 5 mM calcium and 1 μM zinc are required for optimal catalytic activity. Refolded 6 kDa ZBD continued to catalyze the hydrolysis of the fluorogenic peptide substrate over the entire time of the assay. The activity increased in a time-dependent manner (Figure 6B), but the substrate alone showed no digestion. However wild-type matrilysin can completely digest all the substrate within 2 hrs. Although 6 kDa ZBD was able to cleave the peptide substrate and kept its substrate specificity it is much less efficient than the full length active matrilysin. This result strongly suggests that the C-domain, helix-Met-turn-helix, lower part of the active matrilysin is the minimum structural element for substrate recognition and catalytic activities and the N-domain, five-stranded β sheet, upper portion is re1uired for efficient substrate hydrolysis. The protein substrate, CM-transferrin, cleavages seem dependent on the hexamer and octamer formations of 6 kDa in the presence of heparin sulfate and triton X-100 revealed in CM-transferrin zymographic analysis (Figure 7) [27,28]

### **6 kDa ZBD is inhibited by EDTA and synthetic inhibitors, but N-TIMP-2 inhibits ZBD less effective than it does wild type**

This activity could be inhibited by 10 mM EDTA, 1 mM CoCl2 and MMP substrate analogue synthetic inhibitors, SC44463 and BB94, but not by the 24.11 metalloendopeptidase (neprilysin) inhibitor, phosphoramidon, or serine protease inhibitors, such as ZPCK [29]. The concentrations of SC44463 and BB94 required inhibiting 50% of the enzyme activities were about 5 nM. This value is similar to the IC50 for active MMP-7 (Figure 7C). Full length TIMP-1 or N-TIMP-1 did not inhibit ZBD as efficiently as full-length active matrilysin. However, a 25-fold higher concentration of N-TIMP-2 can completely inhibit ZBD (data not shown).

### **Molecular docking of SC44666**

SC44463, (N4-hydroxy-N1-[1 S [(4-methoxphenyl)methyl]-2-(methylamino)-2 -oxoethyl]- 2R-(2-methylpropyl) butane-diamide), A powerful synthetic inhibitor of Matrilysin and collagenases is reported to inhibit ovulation in perfused rat ovaries at 25 nM [30]. From our previous study, sc44463 is a potent inhibitor with IC50 ~10nM [30,31]. We decide to use sc44463 to perform the docking studies for Zinc-Dependent Proteolytic Domain- Helix-Loop-Helix Catalytic Zinc Binding Domain (ZBD) for MMP-7.

**Figure 8:**
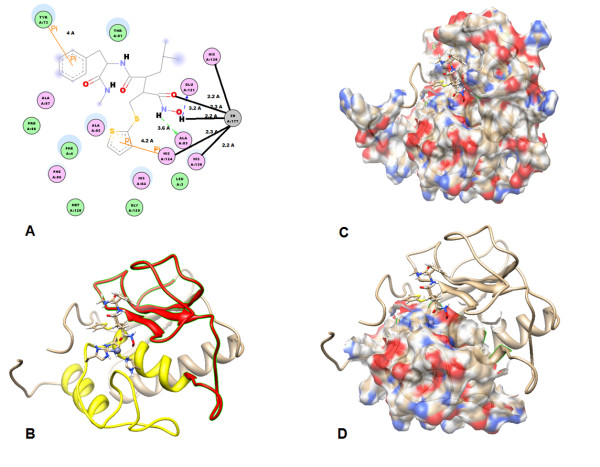
**Molecular docking of SC44463 in Human MMP7.(A)** A 2D diagram of the details of the SC44463 binding environment. The SC44463 was docked onto Human MMP7. Illustration of amino acid contacted to the SC44463 in Human MMP7. SC44463 contacted to TAD (57–114) in the left part (Tyr73, Ala83, Ala85, Ala87) and to HLH (114–173) in the right part (His120, Glu 121, His124, His138, Znic ion). **(B)** Overall illustration of molecular docking of SC44463 in Human MMP7. HLH shown in yellow, TAD shown in red, and other parts of the polypeptide chains in rice white. The binding complex is shown in the standard orientation, i.e. with the cental active center cleft lying horizontally in the paper plane. Zinc ion, three binding histidines and SC44463 are presented with ball and stick model. **(C)** TAD and HLH of binding complex are shown as 30% transparency solid surface colored with surface charge. SC44463 is presented with stick and the complex in rice white ribbon. **(D)** Only HLH of binding complex are shown as 30% transparency solid surface colored with surface charge. It is interesting to notice that the major contact between SC44463 and Human MMP7 are in the HLH area with salt bridge, hydrogen bond and pi-pi interaction. SC44463 is presented with stick and the complex in rice white ribbon.

**Figure 9:**
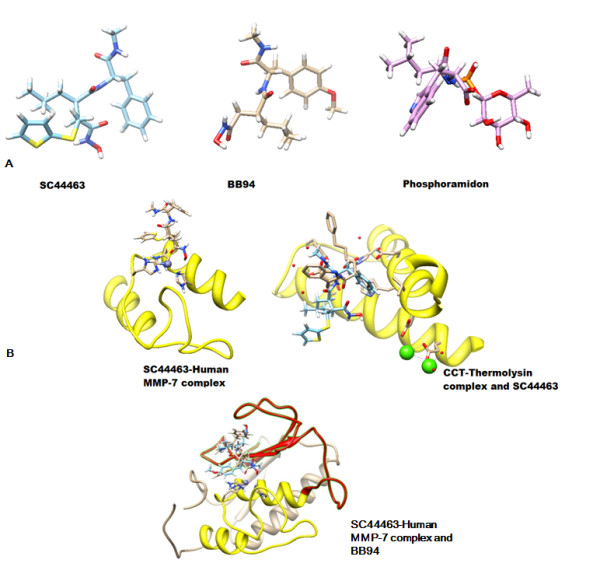
**Comparison docking results of SC44463 in Human MMP7 and in 3.4.24.11 protease and other protease drugs.(A)** The structure of the SC44463, BB94 amd Phosphoramidon is presented as colored stick model. **(B)** The SC44463 was docked onto human MMP7. Illustration of three histidine (His120, His124, His138) of human MMP7 in SC44463-Huaman MMP-7 complex. SC44463 is presented as rice white stick model and HLH of human MMP7 is shown as yellow ribbon. CCT-Thermolysin complex (PDB code: 1THL). The N-(<1-[(2 S)-2-carboxy-4-phenylbutyl]cyclopentyl > carbonyl)-L-tryptophan (CCT) as 3.4.24.11 protease inhibitor is presented as rice white stick model and SC44463 is presented as sky blue stick model. It should be notice that SC44463 could not dock in to 3.4.24.11 protease very well. The huamn MMP7 is presented with ribbon model, HLH shown in yellow, TAD shown in red. The docking result of SC44463-Huaman MMP-7 complex and BB94 shows that the pi-pi and hydrophobic interaction of SC44463 is better than BB94. The SC44463 is presented as sky blue stick model and BB94 is presented as rice white stick model.

Phosphoramidon were not good for docking into MMP7 as negative control for selectivity. SC44463 can successfully dock into MMP7 in multiple residues (in pink) Ala187, Ala184, Phe185, His 124, Ala 183 which contribute up to 70% contact inter phase compared to the whole enzyme. In BB94 case, the results of docking only shown weaker hydrophobic interaction than SC44463 with TAD domain (Figure 8 &9B,3). There is no pi-pi interaction between BB94 and MMP7 as pi-pi interaction between SC44463 and Tyr73 in MMP-7 (Figure 8). The hydroxamate group, HONHCO-CH2C-, of sc44463 contributes to occupy the catalytic zinc to prevent from ionizing H2O molecules. MMP7,a zinc dependent metallopotease, the proteolytic catalytic divalent cation zinc in the active center is involved in the peptide bonds cleavage through the transient electron acceptor for the peptide bond attack by the ionized H2O which Glu-121 donate a pair of electron (Figure 8A).

In this study, we try to define the minienzyme of metzincin super-family. The minienzyme of human MMP7 as ZBD can fold and maintain its catalytic activity. Due to the above description, the mini-enzyme can provide the platform for performing screen or virtual screen to discover broad range of inhibitors. Nevertheless, the discovery of highly selective small molecule protease inhibitors across a wide variety of protease is still achievable. For example, neprilysin (neutral endopeptidase 24.11; NEP) inhibitor, phosphoramidon, obtained by analogs with methyl or ethyl substitutions, was relatively not potent [32]. The docking simulation of SC44463 revealed that this compound blocked the direction of the peptide chain in contact with the active site is in the reverse direction of that seen in complex of stromelysin with synthetic inhibitors [33]. As shown in Figure 8, we found that the SC44463 coordinated the zinc ion and formed pi-pi interaction with side chains of His-124 (HLH) and Tyr-73 (TAD), the oxygen on the main chains of Glu-121 (HLH) of human MMP-7. In addition, Ala-83 (TAD), Ala-85 (TAD), Ala-87 (TAD) donated hydrophobic interaction to SC44463. Thus, the drug docking data of human MMP-7 indicated that SC44463 was a good lead compound for the inhibiting the activity of human MMP-7 through its competitive action with peptide chain pocket. In the discovery and development of metzincin inhibitors, some inhibitors have been approved and successfully targeted to different therapeutic targets in various protease [34]. Our results presented here show that SC44463 was able to suppress the proteolytic activity of human MMP-7 and 6 kDa mini MMP-7. Based on the modeling results, further refinement of the potency and selectivity of SC44463 can be further studied by the modifications of some critical functional groups of SC44463 for rational analog- based searching more potent specific inhibitors for this helix B Met loop-helix C folding domain. Therefore, we speculate that SC44463 can be a good lead compound to develop more potent selective inhibitor for MMP-7.

## **Discussion**

A widely-accepted concept is that the full-length active domain of MMP is the minimum structure required for full catalytic activity and affinity for TIMP. This appears to be true, but the present studies show considerable residual activity in the truncated enzyme. The characteristic consensus motif HEXXHXXGXXH and the Met-turn motif (Table 1), P/Y-XMBX (B: Bulky residues), are the major elements arranged in the common topological structure helix-loop-helix forming the lower half C-terminal portion of the 60 residue domain. [25,35-37]. by adding the upper half five-stranded β sheet (N-domain) (Figure 1), the active center cleft is completed (Figure 8C-D). The exact function of the C-portion which possesses the major catalytic elements is still unknown. It is important to address the molecular and structural function of this helix-Met turn-helix motif, which contains the catalytic zinc element. In evolution, this fundamental building block was augmented by additional gene with the metzincin super-family to give rise ultimately to the present four subclasses. In this report, the folded recombinant ZBD corresponding to the C-portion demonstrates that it possesses the abilities of substrate recognition, catalytic hydrolysis and inhibitor binding (Figure 8A &9). At this point, it will be fair to call this ZBD fragment a zinc binding domain with catalytic function [35].

However, the reasons for the loss of catalytic hydrolysis efficiency compared to full-length active enzyme could be that the five-stranded β sheet (N-domain) is important in contributing hydrophobicity to the active cleft. A common feature for the active center of most enzymes is the non-polar environment in the active center. The hypothesis, as introduced by Cohen *et al.* 1970 and Crosby *et al.* 1970 [38,39], suggested that enzyme active sites becomes basically non-polar after removal of water molecules and that such non-polar sites help in accelerating enzymatic reactions. Applying this hypothetical concept of desolvation Kcat contribution to the situation of this “naked” active center of 6 kDa ZBD, it is not difficult to imagine that the loss of the N-domain five stranded β sheet makes the active center of C-domain completely exposed to a polar environment which does not favor the desolvation process and reduces the catalytic hydrolysis efficiency by two to three orders of magnitude. The contribution of N-domain could be offering the hydrophobicity to the active cleft and helping the desolvation process during the catalytic hydrolysis of substrate. 0.025% Triton X-100 could take the place of the N-domain function, which enhances the hydrophobicity of the active center, helps the desolvation process and enhances the catalytic hydrolysis. Interestingly, ZBD can only digest the intact protein substrate in polymer forms, such as CM-transferrin, but can digest peptides efficiently. One explanation is that this evidence implies that the “naked” active centers of ZBD are buried inside the non-polar environment of Triton X-100 micelle and the peptide substrates can be more easily infused into this micelle and become more accessible for ZBD to hydrolyze than intact protein substrates do. The other explanation is that the helix-loop-helix zinc binding catalytic motif forming the smallest peptide bound cleavage folding unit, however, the rest part of active MMP-7(~19 kDa) facilitates protein-protein interactions to induce conformational changes to expose the cleavable peptide region which could be buried in the intact protein substrates. However, this is purely hypothetical explanation. Another interesting observation regarding the requirement for calcium for activity, which is contradictory to the X-ray crystal structure of active matrilysin [3] needs to be further addressed in the near future.

## **Conclusions**

The helix B-Met loop-helix C folding conserved in metalloprotease metzincin super family is able to facilitate proteolytic catalysis for specific peptide substrate and inhibitor recognition. Triton X-100 plus heparin sulfate is critical for the refolding this 6 kDa helix B-Met loop-helix C domain and also reveal the functional active hexamer and octamer of the mini MMP-7 complex in CM-transferrin zymography. 6 kDa mini MMP-7 through autolysis of proMMP-7 is the smallest metalloprotease in living world.

## **Competing interests**

All authors declare that they have no competing interests.

## **Authors’ contributions**

Wei-Hsuan Yu designs the experiments, collection, and analysis of the experimental data. And writes the manuscript. Po-Tsang Huang and Kuo-Long Lou design and process the docking experiments, write and present the structural relative sections. Shuan-Su C Yu and Chen Lin carried out the molecular cloning, preparation of purified and refolding recombinant 6 kDa mini MMP-7 and drafted the manuscript. All authors read and approved the final manuscript.
