# Correction: A smallest 6 kda metalloprotease, mini-matrilysin, in living world: a revolutionary conserved zinc-dependent proteolytic domain- helix-loop-helix catalytic zinc binding domain (ZBD)

**DOI:** 10.1186/1423-0127-19-87

**Published:** 2012-10-06

**Authors:** Wei-Hsuan Yu, Po-Tsang Huang, Kuo-Long Lou, Shuan-Su C Yu, Chen Lin

**Affiliations:** 1 Institute of Biochemistry and Molecular Biology, National Taiwan University, College of Medicine, Ren-Ai Road, Taipei, Taiwan; 2 Graduate Institute of Oral Biology, National Taiwan University, College of Medicine, Ren-Ai Road, Taipei, Taiwan; 3 NTU-DRCP Lectures and Core for Membrane Proteins, Center for Biotechnology, National Taiwan University, Chang Sing Street, Taipei, Taiwan; 4 Institute of Biotechnology, National Taiwan University, Chang Sing Street, Taipei, Taiwan

## 

There is a major mistake in the order of Figure 5 to Figure 7 in [1]. We replce the Figure 5 and Figure 6 in [1] with new corrected Figures of Figure 1 and Figure 2. We also replace the correct original order of Figure 6 and Figure 7 in [1] with Figure 2 and Figure 3 in this correction. Sorry for the inconveniences!

**Figure 1:**
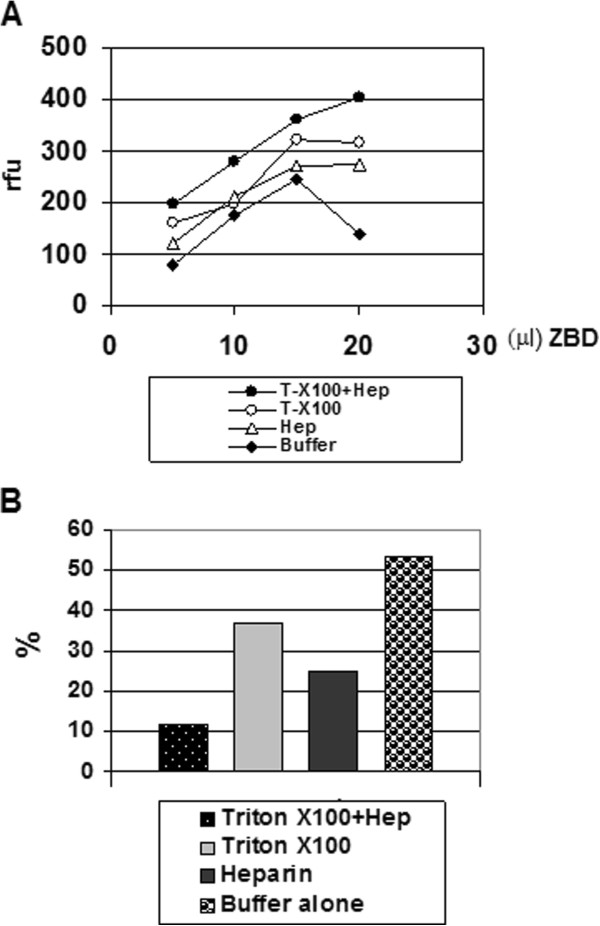
**Combination of 0.05% Triton and 0.2 mg/ml heparin give the optimal refolding activities to cleave the synthetic coumarin-labelled peptide substrate, Mca-Pro-Leu-Gly-Leu-Dpa-Ala-Arg-NH2. ***Panel ***A**: Shows the refolded ZBD activities increased in dose-dependent manner. In the absence of the refolding accessory factors, Triton X-100 and heparin. The significant reduced activities in the high-concentration (> 100 μg/ml) was observed which could be due to autolysis. *Panel ***B**: Under 37°C incubation for 18 hours, Triton X-100 and heparin can prevent the activity loss. (All experiments were repeated at two batch of purification and refolding preparation and data collected from a representative experiments)

**Figure 2:**
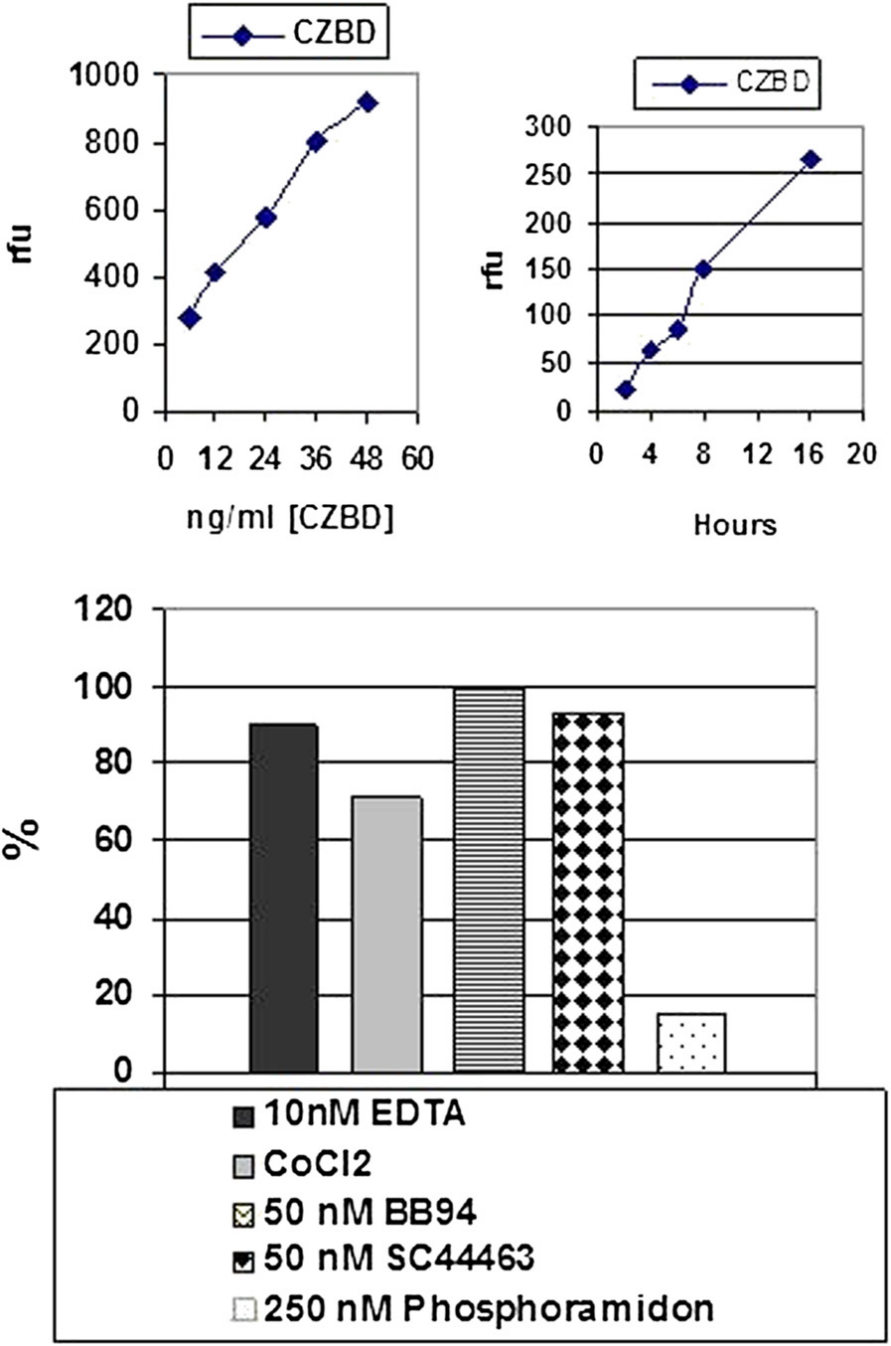
**The polymerization of the 6 kDa ZBD of MMP-7 in pentomer and Octmer demonstrate the significant proteolytic activities towards to the CM-transferrin substrate in CM-transferrin zymographic assay.** 300 μg of craboxylmethylated transferrin (CM-transferrin) was co-polymerized with SDS-PAGE as a substrate gel for analyzing the MMP-7 activities in situ

**Figure 3:**
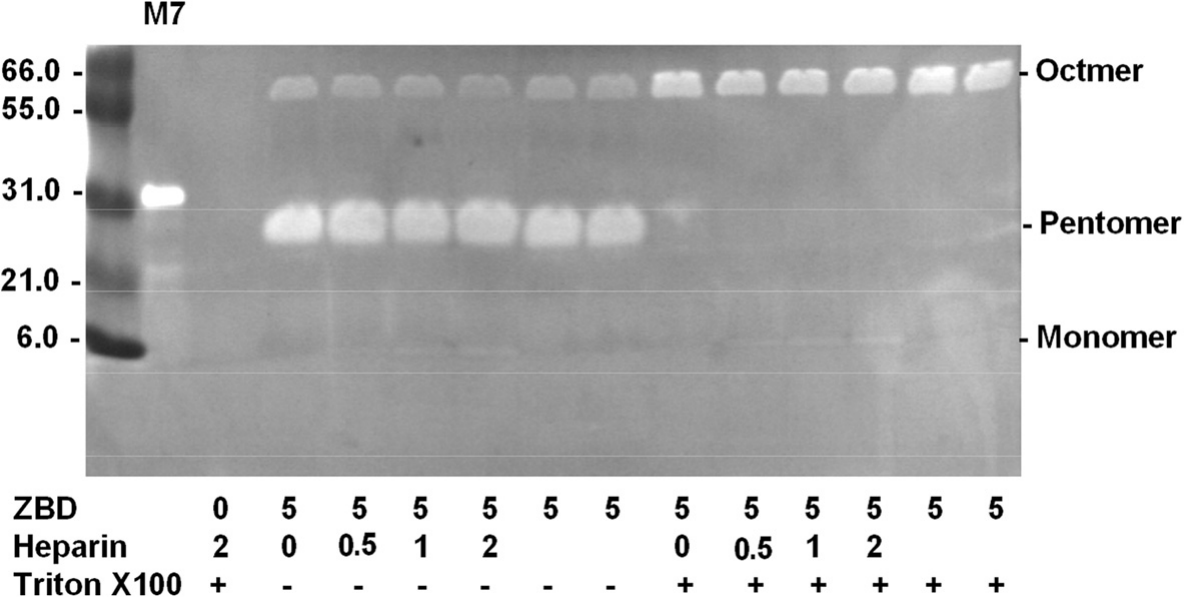
**Mca-Pro-Leu-Gly-Leu-Dpa-Ala-Arg-NH2 assay for characterization of refolded ZBD. ***Panel ***A**: Under the optimized conditions, the refolded ZBD shows increasing enzymatic activity in dose-dependent manner. No significant activity loss was found in the high concentration situation. *Panel*** B**: approximately 6 ng/ml refolded ZBD shows the increasing activity during the time course study and no significant activity loss during overnight incubation. *Panel*** C**: Recombinant ZBD can be inhibited by 10 nM EDTA, 1 mM CoCl2 and synthetic inhibitors, 50 nM BB94 & SC44463 and CoCl2, but not b6 250 nM Phosphoramidon. (All experiments were repeated at two batch of purification and refolding preparation and data collected from a representative experiments)
